# Genetic Characterization of an Endangered Chilean Endemic Species, *Prosopis burkartii* Muñoz, Reveals its Hybrids Parentage

**DOI:** 10.3390/plants9060744

**Published:** 2020-06-12

**Authors:** Roberto Contreras, Liesbeth van den Brink, Boris Burgos, Marlene González, Sandra Gacitúa

**Affiliations:** 1 Centro Regional de Investigación y Desarrollo Sustentable de Atacama (CRIDESAT), Universidad de Atacama, Av. Copayapu 485, 1530000 Copiapó, Chile; 2 Department of Evolution and Ecology, Plant Ecology Group, Universität Tübingen, 72076 Tübingen, Germany; liesbethvandenbrink@hotmail.com; 3 Corporación Nacional Forestal (CONAF), Región de Atacama, Juan Martínez 55, 1530000 Copiapó, Chile; boris.burgos@conaf.cl; 4 Instituto Nacional Forestal (INFOR), Sede Metropolitana y Sede Diaguita, 1760000 Diaguitas, Chile; magonzal@infor.cl (M.G.); sgacitua@infor.cl (S.G.)

**Keywords:** *Prosopis burkartii*, ISSR, SSR, DNA barcode, *trnL* intron, *Strombocarpa* section

## Abstract

The hybridization of *Prosopis burkartii*, a critically endangered endemic species, and the identification of its paternal species has not been genetically studied before. In this study we aimed to genetically confirm the origin of this species. To resolve the parental status of *P. burkartii*, inter-simple sequence repeat (ISSR), simple sequence repeats (SSR) and intron *trnL* molecular markers were used, and compared with Chilean species from the *Algarobia* and *Strombocarpa* sections. Out of seven ISSRs, a total of 70 polymorphic bands were produced in four species of the *Strombocarpa* section. An Multi-dimensional scaling (MDS) and Bayasian (STRUCTURE) analysis showed signs of introgression of genetic material in *P. burkartii*. Unweighted pair group method with arithmetic average (UPGMA) cluster analysis showed three clusters, and placed the *P. burkartii* cluster nested within the *P. tamarugo* group. Sequencing of the *trnL* intron showed a fragment of 535 bp and 529 bp in the species of the *Algarobia* and *Strombocarpa* sections, respectively. Using maximum parsimony (MP) and maximum likelihood (ML) trees with the *trnL* intron, revealed four clusters. A species-specific diagnostic method was performed, using the *trnL* intron Single Nucleotide Polymorphism (SNP). This method identified if individuals of *P. burkartii* inherited their maternal DNA from *P. tamarugo* or from *P. strombulifera*. We deduced that *P. tamarugo* and *P. strombulifera* are involved in the formation of *P. burkartii*.

## 1. Introduction

Species of the genus *Prosopis* L. inhabit the arid and semi-arid areas from the north to the center of Chile [1]. These regions harbor several native taxa: i.e., *Prosopis chilensis* (Molina) Stuntz emend. Burkart, *P. flexuosa var. Flexuosa* DC., *P. flexuosa var. fruticosa* (Meyen) F.A. Roig, *P. alba* Griseb, *P. strombulifera* (Lam.) Benth., *P. tamarugo* Phil. and *P. burkartii* Muñoz [1]. According to the systematic classification of the *Prosopis* genus, the 44 species of the genus *Prosopis* are grouped into five sections that are basically differentiated by the presence, type and distribution of the spines [1,2]. *Prosopis chilensis*, *P. flexuosa* and *P. alba* belong to the section *Algarobia* DC. Emend. Burk, to the *Chilenses* series [1,2]. *P. strombulifera*, *P. tamarugo* and *P. burkartii* belong to the section *Strombocarpa* Bentham. This last section is subdivided into two series, *Strombocarpae* Burkart to which *P. strombulifera* and *P. burkartii* belong, and *Cavenicarpae* Burkart, where *P. tamarugo* is found. Of the *Strombocarpa* taxa, the species *P. tamarugo* and *P. burkartii* and the variety *P. flexuosa var. fruticosa* are endemic to Chile [2,3], and in terms of conservation status, *P. tamarugo* is listed as endangered and *P. burkartii* as critically endangered [4]. *Prosopis burkartii* and *P. tamarugo* inhabit the Tarapacá Region, specifically the Pampa del Tamarugal [5]. Both species are morphologically similar in appearance, foliage and flower; however, *P. burkartii* differs from *P. tamarugo* in its shrub-like structure, intense basal branching, tangled habit and absence of rhizomes [5]. Fruits of *P. burkartii* are bi-spiral and have a septate endocarp, a diagnostic characteristic of *Strombocarpa* section; furthermore, they are compacted and grouped in glomerulus form. On the other hand, fruits of *P. tamarugo* are arranged in an isolated and individual manner, never forming compact masses [5]. In spite of its high risk of disappearing, with a population size of less than 50 mature individuals and located restrictively in the Pampa del Tamarugal (associated with the *P. tamarugo* forest) [6], *P. burkartii* has not been studied in depth for its relation towards other *Prosopis* species and its possible parent species. Burkart [2] noted that the species was a hybrid between *P. tamarugo* × *P. strombulifera*, which was supported by the polypeptide profile [7,8]. However, the hybrid status and parental assumptions of *P. burkartii* have, thus far, not been confirmed through genetic studies.

Natural hybridization is common in plants and plays a very important role in evolution. Understanding its origin requires genetic studies to compare hybrids with their (possible) parental species [9]. To identify hybrids, morphological characteristics were conventionally used. However, some of these characteristics are influenced by the environment and can be difficult to evaluate, resulting in subjective interpretations and eventually incorrect identification [10,11]. More recent studies have used genetic tools to identify natural hybrids and to study hybridization [12], and it has been shown that ISSR (inter-simple sequence repeat) and SSR (simple sequence repeats) markers could provide a high degree of resolution in the relationships and introgression patterns [10,13]. ISSRs are DNA markers that use a single primer composed of dinucleotide, trinucleotide, tetranucleotide and pentanucleotide repetitions; the amplified fragments come from regions of the genome located between two successive, inversely oriented microsatellites [14].

In most plant species, the chloroplast genome is inherited in part or completely from the mother [15,16]. To identify the maternal component of a species, DNA barcoding, which uses a standard chloroplast or nuclear gene or spacer to identify species, can be used [17]. This technique is useful for evolutionary studies and forensic analysis [18,19]. The chloroplast intron *trnL* (UAA) embodies a good region for identifying plant species [20,21]. Although it is not the most variable region of chloroplast DNA, it is the only intron that has a conserved secondary structure with alternating preserved and variable regions [22]. DNA barcoding has been used in several *Prosopis* studies, for example, internal transcribed spacers (ITS) of the nuclear ribosome were used to clarify phylogenetic relationships of populations of *Prosopis* species [23], *matK-trnK*, *trnL-trnF*, *trnS-psbC*, *G3pdh* and NIA markers were used to study diversification and evolution [24], and the *rbcL* gene was used to determinate and validate *Prosopis* species [25]. The objective of this work was to characterize the hybrid status of *P. burkartii* and its possible ancestry genetically, comparing it with native *Prosopis* species from Chile, using ISSR, SSR and DNA barcoding, to clarify the hybrid status of *P. burkartii* and resolve its parental relationship.

## 2. Results

### 2.1. ISSR Analysis

**Figure 1:**
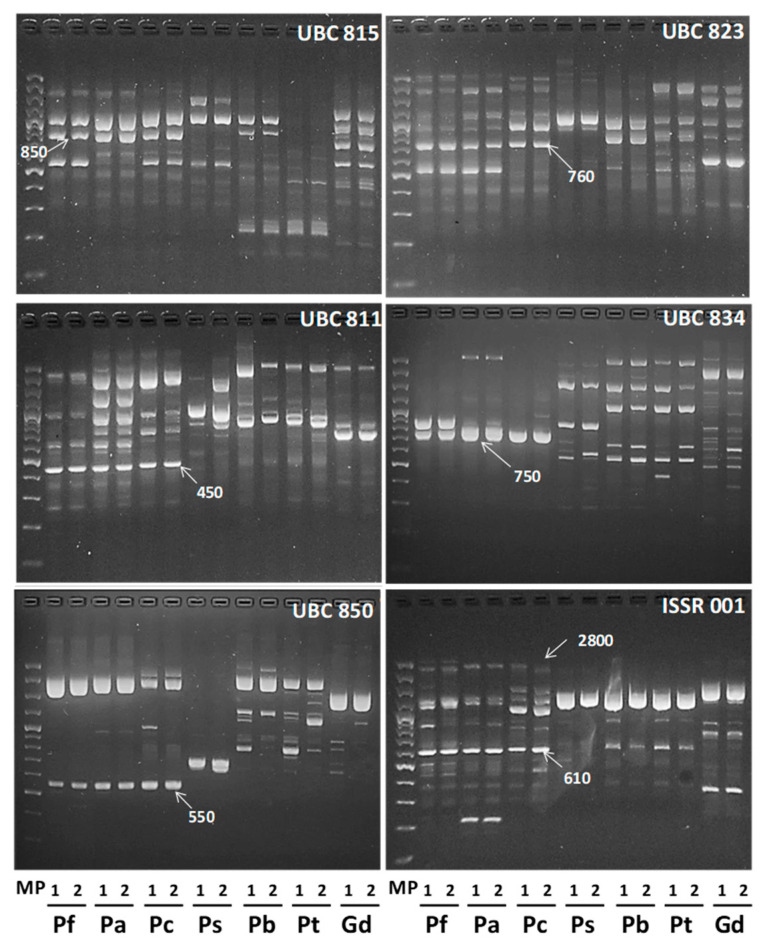
Electrophoresis patterns of six inter-simple sequence repeat (ISSR) markers obtained from DNA samples of *P. flexuosa* (Pf), *P. alba* (Pa), *P. chilensis* (Pc), *P. strombulifera* (Ps1), *P. burkartii* (Pb3), *P. tamarugo* (Pt6) and *G. decorticans* (Gd). Each species has two PCR profile repetitions to check its reproducibility. MP is a marker with a molecular weight between 100 and 3000 bp.

**Figure 2:**
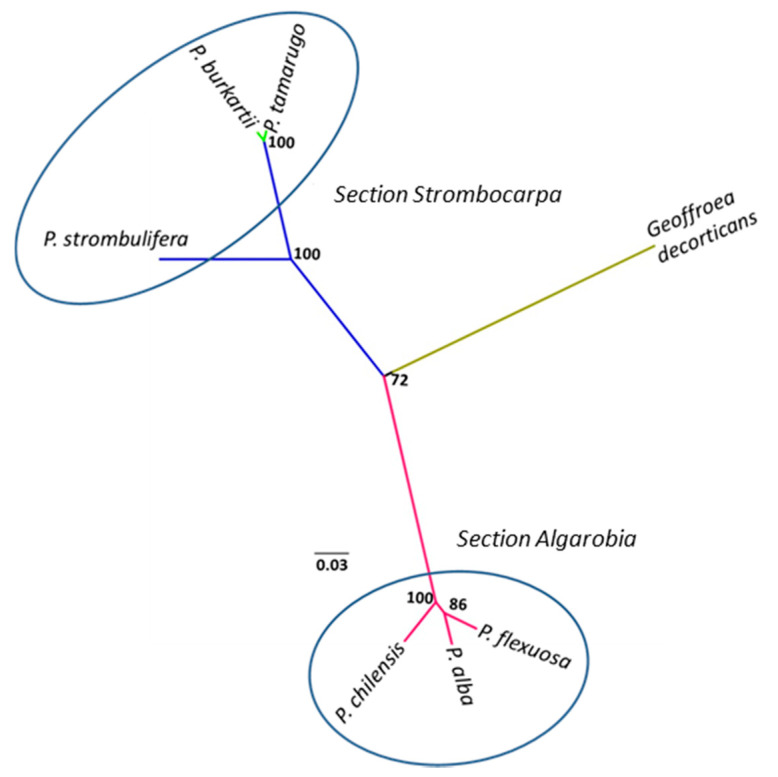
Group analysis of the species in the *Strombocarpa* section (*P. tamarugo*, *P. burkartii* and *P. strombulifera*) and the *Algarobia* section (*P. flexuosa*, *P. chilensis* and *P. alba*), including one outgroup species, *G. decorticans*, based on 12 ISSR markers. The tree was constructed using the UPGMA (unweighted pair group method with arithmetic average) grouping method, and includes the bootstrap values.

To identify the possible *P. burkartii* parental species, 18 ISSR markers were evaluated, of which 12 markers produced defined and robust polymorphic fragments, ranging from 220 to 2800 bp. A total of 235 fragments was produced, of which 232 were polymorphic. A comparative cluster analysis between species in the *Algarobia* section and species in the *Strombocarpa* section was carried out, using the 12 ISSR markers described before. In Figure 1, we show electrophoresis patterns of six ISSR markers that produced fragments exclusively for species of the *Algarobia* section (850 bp with the marker UBC815, 760 bp with UBC823, 450 bp with UBC811, 750 bp with UBC834, 550 bp with UBC850 and 610 bp and 2800 bp with ISSR001), which were absent for species of the *Strombocarpa* section. The cluster analysis (Figure 2) showed three main groups with good bootstrap support (>72), presenting high consistency and support of the clusters. One group was made up by the species *P. strombulifera*, *P. burkartii* and *P. tamarugo* from the *Strombocarpa* section, another group was made up of the species *P. chilensis*, *P. alba* and *P. flexuosa* from the *Algarobia* section and a third group was made up of the species *Geoffroea decorticans* Burkart (outgroup). High reproducibility was observed in most of the ISSR-PCR patterns (84 in total), shown by *P. strombulifera* and *P. burkartii*, except in two of them (UBC811 and UBC850).

**Figure 3:**
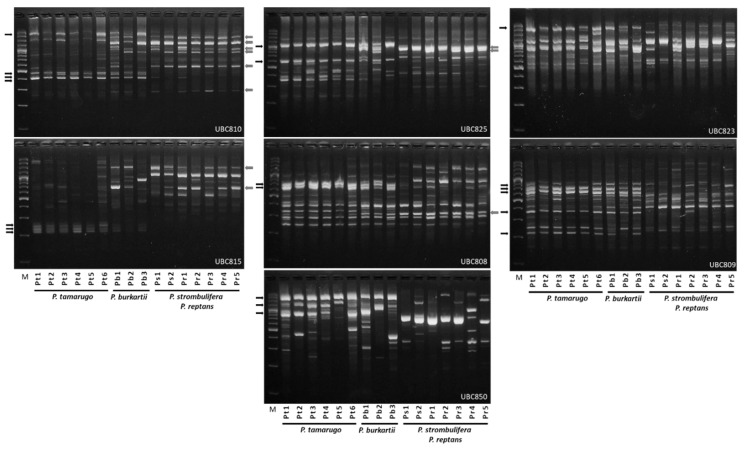
Example of electrophoresis patterns obtained from seven ISSR markers and DNA of six individuals of *P. tamarugo* (Pt1, Pt2, Pt3, Pt4, Pt5 and Pt6), three of *P. burkartii* (Pb1, Pb2 and Pb3), two of *P. strombulifera* (Ps1 and Ps2) and five of *P. reptans* (Pr1, Pr2, Pr3, Pr4 and Pr5). The arrows (at the side of the image) indicate *P. tamarugo* (black) and *P. strombulifera* (grey) PCR fragments, which are shared with at least one individual of *P. burkartii*. M: is a marker with a molecular weight between 100 and 3000 bp.

In Table 1, we present the information obtained from the seven ISSR primers belonging to four species in the *Strombocarpa* section, where we included *Prosopis reptans* Benth. *var. chilensis* Zoellner as a control species. ISSRs produced a total of 78 bands, of which 70 were polymorphic (89% polymorphism). Primers UBC810, UBC815 and UBC850 were 100% polymorphic, while primer UBC823 was 63% polymorphic. Primer UBC825 had the highest number of polymorphic bands (14 bands) and primer UBC823 the lowest number of polymorphic bands (five bands). The highest Rp value was observed with the UBC825 primer (9.33) and the lowest value with the UBC823 primer (3.33), while the highest PIC value was observed with the UBC815 primer (0.46) and the lowest value with the UBC823 primer (0.26) (Table 1). Figure 3 shows the electrophoresis patterns of four species in the *Strombocarpa* section, obtained with seven ISSR markers; next to the figure, indicated with arrows, the PCR bands which *P. burkartii* shares with *P. tamarugo* (20 bands) or *P. strombulifera*/*P. reptans* (11 bands) are shown.

**Figure 4:**
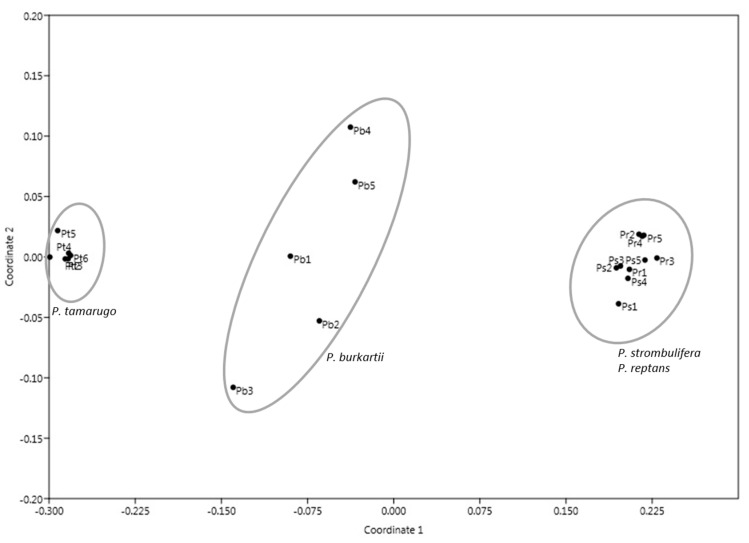
Multidimensional scaling (MDS) that clusters the 21 individuals, using seven ISSR markers.

**Figure 5:**
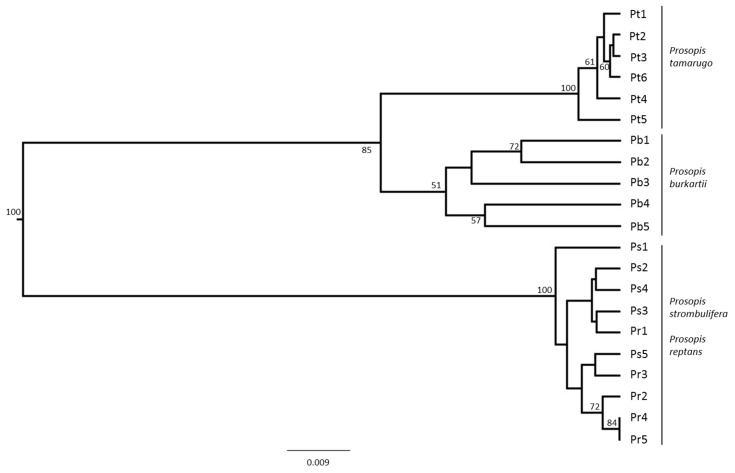
UPGMA dendrogram showing the relationships of individuals of *P. tamarugo*, *P. burkartii*, *P. strombulifera* and *P. reptans*. Bootstrap values greater than 50 percent are indicated.

**Figure 6:**
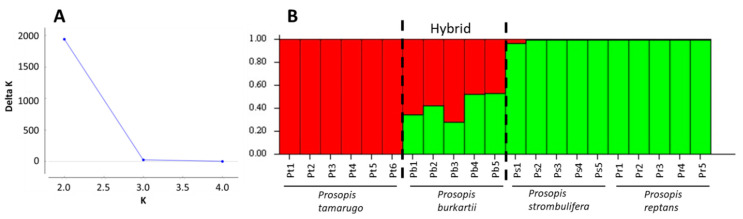
Relationship between number of clusters (K) and corresponding values of Delta K. (**A**). Genetic structure of *P. tamarugo*, *P. burkartii*, *P. strombulifera* and *P. reptans* populations, calculated with the program STRUCTURE. (**B**). The proportion of colors in each bar indicates the probability assigned to each individual.

The multidimensional scaling (MDS) of the species from the *Strombocarpa* section clearly showed the separation between the *P. tamarugo* cluster and the *P. strombulifera*/*P. reptans* cluster, and *P. burkartii* individuals were found at an intermediate (hybrid) position (Figure 4). A UPGMA (unweighted pair group method with arithmetic average) clustering based on the ISSR band pattern showed three similar main clusters with high bootstrap support (>85%). The *P. burkartii* cluster was nested next to the *P. tamarugo* cluster, with good bootstrap support (85%) (Figure 5). A Bayesian analysis using STRUCTURE software, based on the highest probability K = 2 (Figure 6a), identified two main clusters, one with *P. tamarugo* individuals and another one with *P. strombulifera*/*P. reptans*. In addition, a cluster with hybrid genetics, containing *P. burkartii* individuals, was observed (Figure 6b). Furthermore, all the results (Figure 4, Figure 5 and Figure 6) clearly show that *P. strombulifera* and *P. reptans* are related and show a similar genetic structure.

### 2.2. Characterization of the trnL Intron

**Figure 7:**
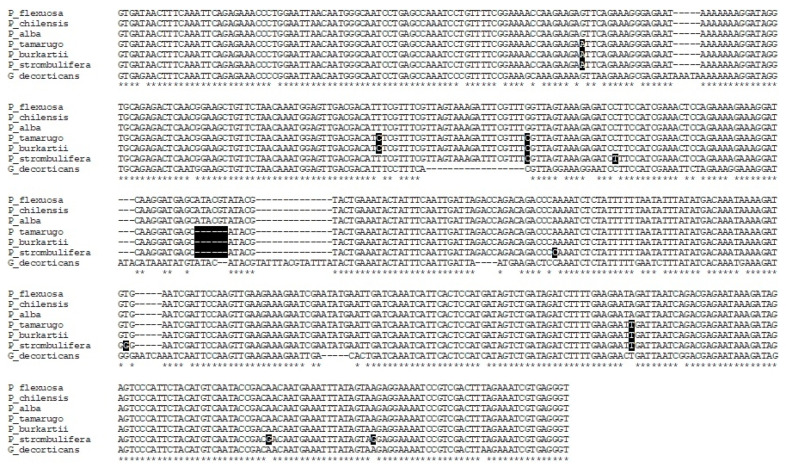
Sequence alignment of the intron trnL (UAA) of the species *P. flexuosa*, *P. chilensis*, *P. alba*, *P. tamarugo*, *P. burkartii*, *P. strombulifera* and *G. decorticans* (outgroup). The polymorphisms (SNPs and Indels) observed among *Prosopis* species were shaded in black. Position 168 shows the SNP C/T.

The sequencing of the trnL intron resulted in a fragment of 535 bp for the species of the *Algarobia* section, and a fragment of 529 bp for the species of the *Strombocarpa* section. In Figure 7, the alignment of the species sequences of the two sections is shown (it also includes the control species, *G. decorticans* as an outgroup), and three different haplotypes were found. One haplotype corresponded with the species of the *Algarobia* section (*P. flexuosa*, *P. alba* and *P. chilensis*), another with *P. tamarugo* and *P. burkartii* and a third with *P. strombulifera*. The species *P. tamarugo* and *P. burkartii* presented four SNPs of difference with the species of the *Algarobia* section, while *P. strombulifera* presented six SNPs of difference with the species *P. tamarugo* and *P. burkartii* and eight SNPs with the species of the *Algarobia* section (Figure 7). Six indels and three SNPs of difference were observed between the species of the *Strombocarpa* section and the species of the *Algarobia* section (Figure 7). *Geoffroea decorticans* had 44 SNPs of difference with the species of the genus *Prosopis*.

### 2.3. Cluster Analysis Using trnL Intron

**Figure 8:**
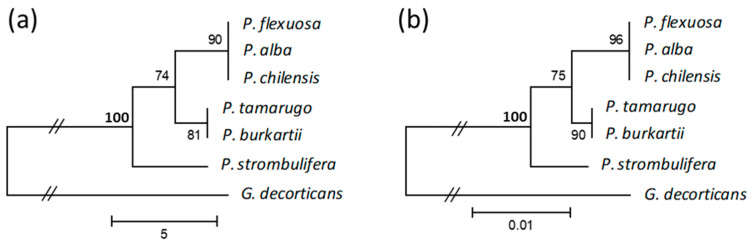
Cluster analysis of the trnL intron sequence data for the species *P. flexuosa*, *P. chilensis*, *P. alba*, *P. tamarugo*, *P. burkartii*, *P. strombulifera* and *G. decorticans* (outgroup). The trees were constructed with the maximum parsimony (**a**) and maximum likelihood (**b**) clustering methods; their corresponding bootstrap values are included in the nodes. The magnitude of the distance of the species *G. decorticans* was shortened to show the two clusters together.

The cladograms constructed from the MP (Figure 8a) and ML (Figure 8b) methods, including the outgroup species *G. decorticans*, showed four main clusters. One cluster was formed by *P. chilensis*, *P. alba* and *P. flexuosa*, from the *Algarobia* section; another cluster was formed by *P. burkartii* and *P. tamarugo* from the *Strombocarpa* section (one species from the *Strombocarpae* series and the other from the *Cavenicarpae* series); another cluster was formed only by *P. strombulifera* from the *Strombocarpa* section; and the last cluster was formed by *G. decorticans*. All the clusters had high bootstrap values (>74), and showed high consistency of the clades.

### 2.4. Parent-Diagnostic cpDNA Markers

**Figure 9:**
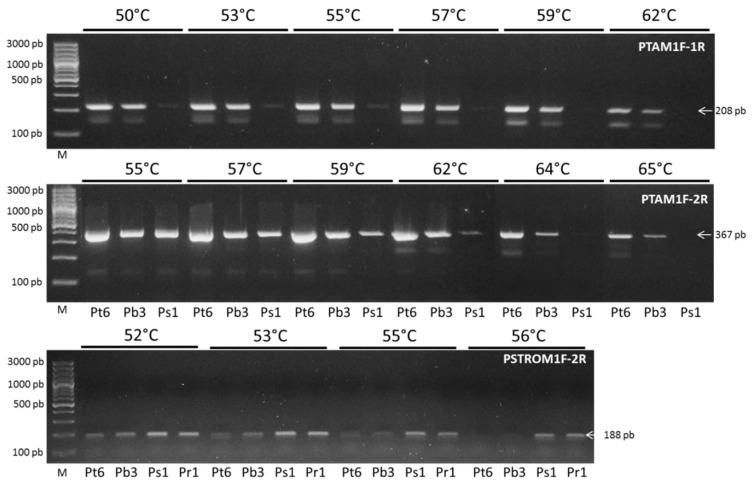
Optimized PCR with specific designed primers for the trnL sequence of *P. tamarugo*, *P. burkartii* and *P. strombulifera* The annealing temperature (Ta) was optimized with DNA samples of Pt6, Pb3, Ps1 and Pr1, by means of a gradient between 50 °C to 65 °C, using the primer pairs PTAM1F-1R, PTAM1F-2R and PSTROM1F-2R. M: is a marker with a molecular weight between 100 to 3000 bp.

**Figure 10:**
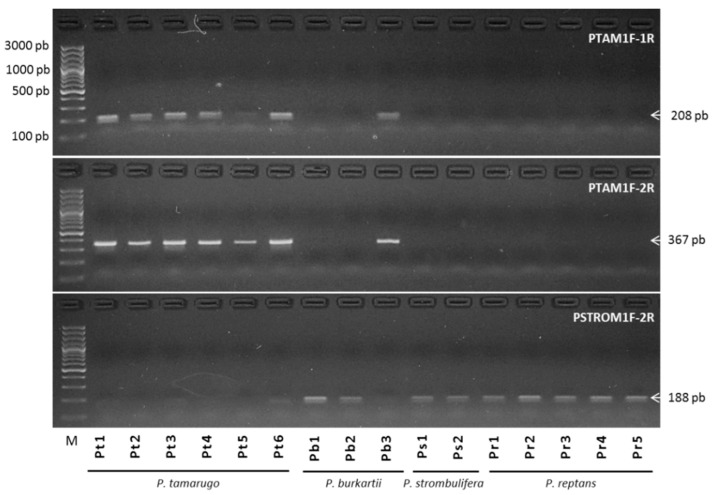
PCR obtained from DNA of six individuals of *P. tamarugo*, three of *P. burkartii*, two of *P. strombulifera* and five of *P. reptans*, using specified PTAM1F-1R, PTAM1F-2R and PSTROM1F-2R primer pairs at an annealing temperature of 62 °C, 65 °C and 56.5 °C, respectively.

We used the *trnL* sequence information for a diagnostic, species-specific PCR method: we designed specific primers using SNPs of cpDNA haplotypes, considering specifically position 168 where the C/T SNP is located (see Figure 7), in order to evaluate the maternal load and optimize the Ta of the primers for the detection of the corresponding haplotype. Our results showed that the primer pairs PTAM1F-1R (at 62 °C) and PTAM1F-2R (at 65 °C), detected only cpDNA of *P. tamarugo* (Pt6) and *P. burkartii*, (Pb3) (Figure 9), but sequences of *P. strombulifera* (Ps1) were not amplified. Alternatively, a pair of PSTROM1F-2R primers was designed to amplify only *P. strombulifera* cpDNA. Our results clearly showed that at Ta of 56 °C, there was no amplification of *P. tamarugo* (Pt6) and *P. burkartii* (Pb3) DNA. However, *P. strombulifera* (Ps1) and *P. reptans* (Pr1) DNA was amplified (Figure 9). This procedure was then used on all the samples, with special focus on the amplification of the *P. burkartii* individuals. The results (Figure 10) showed amplification in all samples of *P. tamarugo* (Pt1 to Pt6) with the primer pairs PTAM1F-1R and PTAM1F-2R, while none of the samples of *P. strombulifera* (Ps1, Ps2) and *P. reptans* (Pr1–Pr5) showed amplification. It should be noted that DNA in the samples Ps3, Ps4 and Ps5 was not amplified either, but those samples were not included in the figure due to the reduced space on the gel. Surprisingly, DNA in some of the *P. burkartii* samples was amplified and produced the expected fragment (Pb3 and Pb5), while DNA in the others did not (Pb1, Pb2 and Pb4), as shown in Figure 10. We performed the same procedure with the PSTROM1F-2R primers, in order to confirm the results obtained by the PTAM1F-1R and PTAM1F-2R markers. Indeed, in all *P. strombulifera* and *P. reptans* samples, the expected fragment size was amplified, while in *P. tamarugo* samples, no fragments were amplified. Therefore, we confirmed the differences in the five *P. burkartii* samples with these primers. We concluded that *P. burkartii* individuals, Pb3 and Pb5, have *P. tamarugo* as their maternal parent, whereas *P. burkartii* individuals, Pb1, Pb2 and Pb4, have *P. strombulifera* as their maternal parent.

### 2.5. Species Diagnostic by SSR-PCR Markers

**Figure 11:**
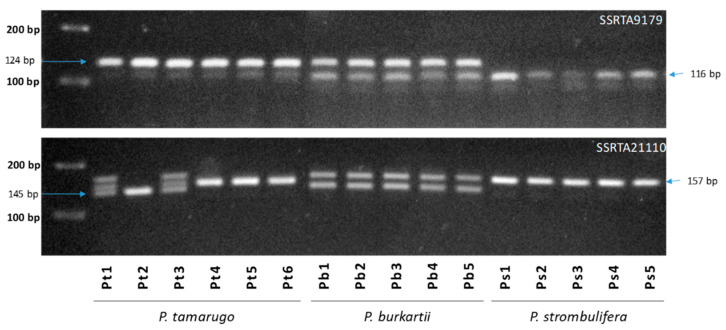
Banding patterns of SSR-PCR of markers SSRTA9179, SSRTA21110 and SSRTA10814 on an agarose gel. The lanes containing six individuals of *P. tamarugo* (Pt1–Pt6), five individuals of *P. burkartii* (Pb1–Pb5) and five individuals of *P. strombulifera* (Ps1–Ps5).

According to Figure 11, two nuclear SSR markers (SSRTA9179, SSRTA21110) showed different bands for the *P. tamarugo*, *P. burkartii* and *P. strombulifera* species. The SSRTA9179 marker showed a band, with a fragment of 124 bp in all *P. tamarugo* individuals, and a band with a fragment of 116 bp in all *P. strombulifera* individuals, while all *P. burkartii* individuals showed both fragments. Moreover, the SSRTA21110 marker showed a band with a fragment of 157 bp in all *P. strombulifera* individuals, and a band with a fragment of 145 bp in three *P. tamarugo* individuals (Pt1–Pt3), while all *P. burkartii* individuals could be seen in both fragments. 

## 3. Discussion

Although there was evidence that *P. burkartii* could have a hybrid origin involving *P. tamarugo* and *P. strombulifera*, based on morphological descriptions and electrophoresis studies of seed proteins [2,7,8], there is no genetic study to corroborate the hybridization and possible parent species thus far. Our results from the ISSR markers demonstrated a high support for the separation of two large clusters, the *Algarobia* section and the *Strombocarpa* section. Previous work has already shown that the species that make up each section are highly differentiated [26,27]. Similarly, the cluster analyses showed that the species of the *Strombocarpa* section are clearly different from the *Algarobia* section [28]. The electrophoresis patterns of the ISSR primers used in our study revealed the diagnostic markers of the species of the *Strombocarpa* and *Algarobia* section. A study carried out with RAPD primers found specific markers for species of the two sections as well [29]. Between these studies and our results, there is sufficient evidence that it would be unlikely to find ancestors of *P. burkartii* within the species from the *Algarobia* section. Therefore, for upcoming analyses, only species from the *Strombocarpa* section could be used to find potential parental candidates. It should also be noted that between species of the *Algarobia* section and species of the *Strombocarpa* section no hybrid formation has been reported [26].

The ISSR markers showed a high number of polymorphic bands when looking at the (21) individuals from the *Strombocarpa* section (*P. tamarugo*, *P. burkartii*, *P. strombulifera* and *P. reptans*), and specific primers (UBC810, UBC825, UBC815 and UBC850) showed a high content of polymorphic information. Of the various genetic tools used to study hybridization in plants, the ISSR marker is one of the simplest molecular methods that can be used for comparative analysis of possible hybrids and parental species [10,30,31,32]. The electrophoresis patterns of seven ISSR primers showed up to 19 bands shared between *P. burkartii* and the species *P. tamarugo*, *P. strombulifera* and *P. reptans*. However, more bands were shared with *P. tamarugo* than with the other species. The electrophoretic profile of seed proteins identified a total of 31 bands in *P. strombulifera*, and 33 in *P. tamarugo*, while the profile of *P. burkartii* presented 40 bands in total, of which 20 were shared by all three species [33]. In addition, that study indicates that most of the seed proteins of *P. burkartii* showed a similar pattern to that of *P. tamarugo*, which agrees with our results, as we found the largest counts of shared ISSR bands with *P. tamarugo*.

A UPGMA cluster analysis showed that the *P. burkartii* cluster is nested with the *P. tamarugo* cluster and an MDS multivariate analysis places it in an intermediate position between *P. tamarugo* and *P. strombulifera*/*P. reptans*. The genetic affinity between *P. burkartii* and *P. tamarugo* is consistent with the clustering analyses observed in other studies, where a high number of shared protein bands (obtained by electrophoretic analysis) between these species was observed [8]. According to the MDS and STRUCTURE analyses, signs of introgression of genetic material in *P. burkartii* are evident, confirming its hybrid status (*P. tamarugo* × *P. strombulifera*). However, according to the same analyses there might be differences in introgression levels between the analyzed samples. Additionally, our results showed, through MDS, UPGMA clustering and STRUCTURE analysis, that *P. reptans* and *P. strombulifera* could be considered to be one single species. This is corroborated by several studies that previously proposed that *P. reptans* and *P. strombulifera* are the same species, and could be considered subspecies or variants. This was validated by morphological classification [2], enzymatic studies [34], genetic studies [8] and morphological-biochemical studies [28]. However, *P. reptans* has not been found in the Pampa del Tamarugal, while *P. burkartii* is an endemic species inhabiting a restricted area, located mainly in the Pampa del Tamarugal, in the Atacama Desert (Tarapacá Region). It is distributed over approximately 1395 km^2^ [6], and only 50 specimens have been found [35], occupying the same ecological niche as *P. tamarugo* and *P. strombulifera* [5]. From the samples collected during this study (within the Pampa del Tamarugal) a high association was observed between *P. burkartii* and *P. tamarugo*, growing physically close to each other. This could help the processes of introgression, as fertilization of *P burkartii* flowers by *P. tamarugo* pollen can easily take place [33]. Introgression with *P. strombulifera* genetic material was unlikely, as very few individuals were observed in the sampling areas.. According to Burghardt [8], polypeptide analyses suggest that the placement of *P. burkartii* in the *Strombocarpae* series needs to be revised. Due to the affinity of bands between *P. burkartii* and *P. tamarugo*, they suggested that *P. burkartii* would belong to the *Cavenicarpae* series and not to the *Strombocarpae* series [2]. This is corroborated by the amplificated fragments, obtained by the ISSR markers, in our study.

Interspecific hybridization occurs commonly and naturally between species in the *Algarobia* section that occupy the same geographical area [26]. However, the ability to hybridize is not present in all species of this section [24]. Thus far, there are no other records of species hybridization in the *Strombocarpa* section, except for *P. burkartii*. The results we obtained with the ISSR and SSR markers confirm the natural hybridization of *P. burkartii*. However, the various molecular tools available, such as fluorescence in situ hybridization (FISH) and genomic in situ hybridization (GISH) [36,37], could contribute to verify gene or chromosome segment introgression.

The ability of the cpDNA barcode to identify parental species of hybrids is well documented; for example, the maternal parent of *Rhizophora* L. hybrids was revealed with data from mangrove chloroplast regions [38]. Additionally, in species of the genus *Spondias* L. *matK*, *rbcL* and *trnH-psbA* markers were used [39]. Moreover, in interspecific hybrids of Annona L. species, *rbcL* and *matK* markers exposed the maternal species [40]. In addition, “diagnostic” DNA markers are commonly used in species validation studies [41]. In our case, the intron *trnL* was able to amplify six species of both *Strombocarpa* and *Algarobia* section, and showed important polymorphic differences. While it is true that analyses can be strengthened by increasing the quantity of genes and/or chloroplast or nuclear spacers, this study also tested *matK* [42], *rbcL* [43] and *ITS* [44] markers, which did not amplify (or showed weak amplification and/or multiple amplifications) in *P. tamarugo*, *P. burkartii* and *P. strombulifera*.

Even though *P. burkartii* and *P. tamarugo* species belong to the *Strombocarpa* section, our cluster analyses show them to be stronger related to *P. alba*, *P. chilensis* and *P. flexuosa* (*Algarobia* section) and separates them from *P. strombulifera*, the type species of the *Strombocarpa* section. This result is contradictive to what is currently established; the species of the *Strombocarpa* section are separated from the *Algarobia* section [24,26,27,28]. Due to the fact that information of one region only (*trnL*) might not able to separate the *Strombocarpa* and *Algarobia* sections, we recommend to gain more information with other regions and markers. As indicated before, we were unable to obtain more sequences with the universal markers for chloroplast (*matK*, *rbcL*) and nuclear (*ITS*) region for the species from the *Strombocarpa* section. However, Catalano et al. [24] observed the separation of the section *Strombocarpa* and *Alagarobia*, based on data gained with three markers (*trnS-psbC*, *G3pdh* and *NIA*).

When sequencing the *trnL* intron of *P. burkartii,* and comparing the sequence alignment with the rest of the *Algarobia* and *Strombocarpa* species, we observed that *P. burkartii* and *P. tamarugo* had the same haplotype. Therefore, we could believe that *P. burkartii* has *P. tamarugo* as their maternal parent. However, these results were obtained from only one individual, and we decided to extend the analysis to the other samples. For this, we used a simple and less expensive method without the need for DNA sequencing: identifying cpDNA from SNP. SNPs are a variation of the DNA sequence that affect only one base (A, G, T and C) of the genome, and have become important genetic markers for animal or plant species [45]. They have been used for genotyping plant material, e.g., through the Allele-Specific PCR method. This technique is based on the design of a primer that extends to the 3′ end where the SNP is located, detecting the nucleotide that corresponds to the specific SNP site [46,47]. In the sequences of the intron *trnL* obtained from the *Algarobia* and *Strombocarpa* section, we found a (C/T) SNP in position 168, which differentiates *P. burkartii* and *P. tamarugo* from the rest of the species (Figure 8). Using this SNP and specific primers (PTAM1F-1R, PTAM1F-2R and PSTROM1F-2R), we could check for the maternal parent of *P. burkartii* in the other collected samples. In all samples, collected (21) from the *Strombocarpa* section, the primer pairs (PTAM1F-1R, PTAM1F-2R) were able to detect the cpDNA haplotype of *P. tamarugo*, while the primer pair (PSTROM1F-2R) was able to identify the cpDNA haplotype of *P. strombulifera* and *P. reptans*. We confirmed that some individuals have *P. tamarugo* (Pb3 and Pb5) and others *P. strombulifera* as maternal parent (Pb1, Pb2 and Pb4), by using the combination of those primers on five samples of *P. burkartii*. It is believed that cpDNA is predominantly inherited by the mother in higher plants, as 80% of angiosperm genera show a strictly maternal cpDNA inheritance [48]. However, there are cases in which it can also be transmitted in a biparental or paternal manner [49]. In our study, double amplification has not been observed in the *P. burkartii* samples, both with PTAM and PSTROM primers. Additional molecular techniques and sequencing more individuals of *P. burkartii* and their parents are recommended to confirm our findings, and provide data of interspecific hybridization and the level of introgression. We believe that these results from the SNPs and the bands shown by the SSRs markers in our work confirm the hybrid status of *P. burkartii*.

## 4. Materials and Methods

### 4.1. Plant Material

**Figure 12:**
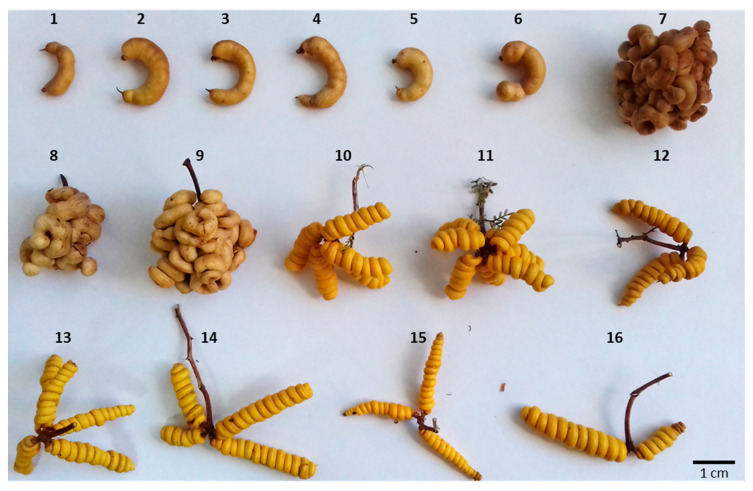
Fruits of *Prosopis* tamarugo (Pt1 (1), Pt2 (2), Pt3 (3), Pt4 (4), Pt5 (5), Pt6 (6)), P. burkartii (Pb1 (7), Pb2 (8), Pb3 (9)), P. strombulifera (Ps1 (10), Ps2 (11)) and P. reptans (Pr1 (12), Pr2 (13), Pr3 (14), Pr4 (15), Pr5 (16)).

Fresh leaves of six species from the genus *Prosopis* were collected between April 2018 and March 2019; three species from the *Algarobia* section and three species from the *Strombocarpa* section. The species *P. chilensis* was collected by the Forest Institute of Chile (INFOR) in the Metropolitan Region, while CRIDESAT collected species of *P. tamarugo*, *P. strombulifera* and *P. burkartii* in the Pampa del Tamarugal, and *P. alba* and *P. flexuosa* in the Copiapó Valley. In order to compare with another species in the *Strombocarpa* section, we include a seventh species, *P. reptans*, which has only been found in the Atacama Region [50], and was collected at the same location as first found by Zöllner and Olivares [50] (Km 837 of the Pan-American Highway 5 north). Additionally, a genotype of *P. burkartii* from San Pedro de Atacama (Antofagasta Region, collected by CONAF) was used, which was grown from seed to seedling stage. According to CONAF, there are records of four adult individuals of *P. burkartii* in San Pedro de Atacama. The species of *Geoffroea decorticans* (Gd; 27°20′39.3 “S 70°21′46.0 “W), belonging to the same family as *Prosopis* (Fabaceae or Leguminosae), was included as an external (out-) group. Taxonomic identification of the species was done using keys and descriptions [2,51]. The fruits of the sampled species of the *Strombocarpa* section are shown in Figure 12, and Table 2 gives an overview of the collected samples, their geographical location and the registration number (Index Herbarium Code) given by the EIF herbarium of the Department of Forestry and Nature Conservation (Faculty of Forestry Sciences, University of Chile).

### 4.2. DNA Extraction

DNA was extracted from fresh leaves, using a modified CTAB (cetyl trimethylammonium bromide) method described by Contreras et al. [52]. Fine fresh leaf powder (100 mg) was obtained after grinding the tissue with liquid nitrogen. The powder was placed in 2 mL tubes and mixed with 700 µL of extraction buffer [Tris-HCl 100 mM, pH 8.0; NaCl 1.4 M; EDTA 20 mM; 2% (*w*/*v*) CTAB], supplemented with 1% of β-mercaptoethanol, 14 µL of 5% Sarkosyl, 0.045 g of D-sorbitol (MW 182.17 g/mol), 2% PVP-40 and 4 µL proteinase K (10 mg/mL), and preheated to 65 °C. The samples were vigorously shaken for 10 s and incubated for 1 h at 65 °C, this step was repeated three times to improve the mixture. The water phase was recovered by centrifugation at 14,000 rpm for 10 min, and then mixed with an equal volume of chloroform-isoamyl alcohol (24:1). The mixture was gently swirled for 2 min and centrifuged at 14,000 rpm for 10 min. The upper phase was transferred to a new tube and treated with 5 µL RNase A (100 µg/mL) and incubated at 37 °C for 30 min. The extraction was mixed with two thirds volume of isopropanol at −20 °C and gently swirled 30 times. A volume of 600 µL of the mixture was transferred to a HiBind DNA^®^ minicolumn with a collecting tube (Omega Bio-Tek) and incubated for 2 min at room temperature. The minicolumns were centrifuged for 1 min at 10,000 rpm and the purified liquid was discarded. A precipitation solution of 600 µL 70% ethanol and ammonium acetate (10 mM) was added to the minicolumns and centrifuged again for 1 min at 10,000 rpm. The liquid was discarded and the precipitation step was repeated. To ensure that all the ethanol was removed, the sample was centrifuged for 2 min at 14,000 rpm, and the collection tube was replaced with a new 1.5 mL tube. To elute the DNA, 40 µL of Tris-EDTA buffer (TE) was added to the minicolumns, which were then incubated at 37 °C for 20 min, followed by a 2-min centrifugation at 14,000 rpm; the DNA eluate was then stored at −20 °C for further analysis. The quality and concentration of the total DNA was checked with a Colibri micro volume spectrophotometer (Titertek Berthold, Germany) at 260, 280 and 230 nm; in addition, the integrity of the genomic DNA was checked on a 0.7% agarose gel.

### 4.3. ISSR Amplification

We used 18 UBC (University of British Columbia) markers for the ISSR amplification: UBC802, UBC807, UBC808, UBC809, UBC810, UBC811, UBC813, UBC815, UBC816, UBC818, UBC821, UBC823, UBC825, UBC834, UBC850, UBC880, UBC856 and ISSR001 [53,54], of which 12 were selected that provided higher numbers of fragments and better reproducibility. The 20 µL PCR solution consisted of: 10 µL Master Mix SapphireAmp 2× (Takara, Clontech, USA), 5 µL ISSR primer (5 µM), 1 µL genomic DNA (5 ng/µL) and 4 µL nuclease-free water. Amplifications were performed in a MultiGene Optimax thermal cycler (Labnet) using the following protocol: an initial step of 5 min at 94 °C, 45 cycles of 30 s at 94 °C, 45 s at 52 °C and 2 min at 72 °C, followed by a final extension step of 6 min at 72 °C. The amplification products were separated by agarose gel electrophoresis (1.2%) and colored with GelRed^TM^ (10,000×) at the ratio of 10 µL/100 mL of gel (with Tris-borate-EDTA at 0.5×). The electrophoresis run was performed at 100 V for 110 min. The gel was then placed in a UV trans illuminator (Vilber Lourmat, France) and photographed with a digital camera (Canon, SX160 IS. USA).

### 4.4. TrnL Amplification and Sequencing

For the amplification of the *trnL* intron (UAA) the primers forward A49325 5′-CGAAATCGGTAGACGCTACG-3′ and reverse B49863 5′-GGGATAGGGACTTGAAC-3′ described by Taberlet et al. [21] were used. The 24 µL PCR solution was prepared as follows: 12 µL Master Mix SapphireAmp 2× (Takara, Clontech), 1.5 µL forward (5 µM), 1.5 µL reverse (5 µM), 6 µL genomic DNA (5 ng/µL) and 3 µL nuclease-free water. Amplification was performed in a MultiGene Optimax thermal cycler (Labnet) using the following conditions: an initial step of 5 min at 95 °C, 35 cycles of 45 s at 95 °C, 45 s at 55 °C and 2 min at 72 °C, followed by a final step of extension of 6 min at 72 °C. The amplified products were separated and visualized as described in the “ISSR Amplification” section. The PCR amplicons were cut from the gel and purified with the Wizard^®^ SV Gel and PCR Clean-Up System (Promega) kit, according to the manufacturer’s recommendations. The purified PCR product was then shipped to Macrogen Inc. for sequencing using ABI3730XL equipment. (Seoul, South Korea).

### 4.5. ISSR Analysis

In each individual (21), the ISSR-PCR fragments were considered as an independent locus, which were analyzed as presence (1) or absence (0) of bands. We obtained the total number of ISSR bands (TNB), percentage of polymorphic bands (P%) at 99%, the number of different genotypes (NG), resolving power (Rp) and the number of private bands (NPB). The resolving power (Rp) of a primer was calculated as Rp = Σ Ib, where Ib (band information) takes the value of 1 − [2 × (0.5 − p)], with p being the frequency of the lines the band contains [55]. The polymorphic information content (PIC) was estimated as PIC = 2p (1 − p) [56].

The similarity index was calculated from presence-absence matrix of the bands. This matrix was used for the construction of a dendrogram, applying unweighted pair group method with arithmetic average (UPGMA). The bootstrap values with 1000 repetitions were obtained with the Winboot program [57] to test the robustness of the groups. The dendrograms were built with Phylip 3.6 software [58] and edited using FigTree 1.4.0 [59]. Additionally, a multivariate analysis by Multidimensional Scaling (MDS) was performed using the PAST program [60].

The genetic structure was determined by a Bayesian analysis, using STRUCTURE v.2.3 software [61]. The optimal number of subpopulations (K) was identified after five independent runs for each K value oscillating between 1 to 5, with a burn-in period of 10,000 replicates followed by 20,000 replicates of Markov Monte Carlo Chains (CMMC). We examined the delta K criteria (ΔK) to find the optimal K values [62], which were entered into the Structure Harvester program [63].

### 4.6. Barcode Analysis

The forward and reverse sequences were edited with the software Chromas Pro v1 (Technelysium Pty, Ltd.). The DNA sequences of both ends were assembled, using the DNA Baser Sequence Assembler v4.10 software (Biosoft 2012). Sequence editing was done, using the automatic analyses for editing “contig” with default program parameters. To characterize the sequences and observe differences in SNPs (Single Nucleotide Polymorphisms) and indels, multiple sequence alignments were performed using MEGA 6 software [64] and the ClustalW program [65]. The sequences were deposited in Genbank with the following codes: *P. flexuosa* (MK450531), *P. chilensis* (MK450532), *P. alba* (MK450533), *P. tamarugo* (MK450534), *P. burkartii* (MK450535), *P. strombulifera* (MK450536) and *G. decorticans* (MK450537). Cluster analyses were performed using the MEGA 6 program in order to illustrate the differences between species and clusters. Trees were generated using maximum parsimony (MP) and maximum likelihood (ML) Bayesian methods. All cluster analyses were performed with a bootstrap support of 1000 replicates.

### 4.7. Specific Primer Design and PCR Optimization

Specific primers were designed, using the polymorphic information of *trnL* sequences obtained from *P. tamarugo* (MK450534), *P. burkartii* (MK450535) and *P. strombulifera* (MK450536). The sequences were aligned with MEGA 6 and “Clustal W” software. Using the Oligo^®^ software, primer pairs were designed with a mismatch in the 3′ SNPs of *P. tamarugo* and *P. strombulifera*, in order to identify the chloroplast DNA (cpDNA) of the specific individuals. The primer pairs to identify chloroplast DNA of *P. tamarugo* species were PTAM-1F (5′-AAATGGAGTTGACGACATC-3′)/PTAM-1R (5′-TCAACTTGGAATCGATTCA-3′) with a 208 bp product and PTAM-1F/PTAM-2R (5′-GTCGACGATTTCCTCTT-3′) with a 367 bp product. The primer pair for identifying cpDNA of *P. strombulifera* species was PSTROM-1F (5′-ATGGAGTTGACGACATT-3′)/PSTROM-2R (5′-CCATCTTTTATTGTCATATA-3′) with a product of 188 bp. The PCR solution contained: 8 µL Master Mix SapphireAmp 2× (Takara, Clontech), 0.8 µL forward (5 µM), 0.8 µL reverse (5 µM), 3.2 µL genomic DNA (5 ng/µL) and 3.2 µL nuclease-free water. Amplification was performed in a MultiGene Optimax thermal cycler (Labnet) with the following protocol: an initial step of 4 min at 94 °C, 30 cycles of 45 s at 94 °C, 45 s at annealing temperature (Ta, see below) and 1 min at 72 °C, followed by a final extension step of 5 min at 72 °C. The amplification products were separated and visualized as described in the “ISSR Amplification” section. To determine the optimal Ta, a PCR gradient ranging from 50 °C to 65 °C was used with DNA from Pt6, Pb3, Ps1 and Pr1 samples. With the specific primer pairs already optimized, PCRs were then performed on the rest of the samples from the *Strombocarpa* section.

### 4.8. Fingerprinting by SSR–PCR Reaction

Two SSR markers develop from *P. Tamarugo* described by Contreras et al. [66] were selected. The SSR primer pairs SSRTA9179 (F: TGAATTGTATGGAAATACGACTCTG and R: TCATTGGCCCTTGTAGTTGA; motif TTTC; accession MT136897) and SSRTA21110 (F: TGGTTGGCTCAAAAGTGAAA and R: TGTGAGAAGCAAGTCCTCGTT; motif TG; accession MT136909) were used. PCRs were carried out in a total volume of 16 µL that contained 8 µL of SapphireAmp Fast PCR 2× Master Mix (Takara-Clontech, USA), 3.2 µL of genomic DNA (5 ng/µL), 0.8 µL of each primer (forward and reverse, at a 5 µM concentration), and 3.2 µL of nuclease-free water. PCR amplification was conducted in a Labnet MultiGene OptiMax Thermal Cycler under the following conditions: DNA was denatured at 94 °C for 3 min, followed by 45 cycles of 98 °C for 5 s, Ta of 59 °C for 5 s, 72 °C for 40 s and a final extension at 72 °C for 4 min. The PCR products were analyzed by electrophoresis on 2% agarose gels stained with GelRed DNA stain (10,000×, Biotium). The band sizes were approximated based on 100 bp DNA ladder (Thermo Fisher, Waltham, MA, USA)

## 5. Conclusions

In conclusion, we could confirm the natural hybrid status of *P. burkartii*, from the MDS, UPGMA and STRUCTURE analyses based on ISSR fragment data, SSR-PCR, and the results of *trnL* intron-specific PCR tests. We could also deduce that *P. tamarugo* and *P. strombulifera* are likely to be involved as source species. This is the first time that ISSR, SSR and *trnL* sequence data are provided for *P. burkartii*, to clarify its hybrid status and possible parental status. There is a high diversity of individuals with different maternal components, distributed in the Pampa del Tamarugal (Tarapacá Region) and in San Pedro de Atacama (Antofagasta Region). Unambiguously, this study indicates the large commitment that government institutions should provide to this species, which requires better legal protection and urgent measures for its conservation.

## Figures and Tables

**Table 1:** ISSR primer information using *P. tamarugo*, *P. burkartii*, *P. strombulifera* and *P. reptans* species. Total number of bands (TNB), number of polymorphic bands (NPB) at 99%, percentage of polymorphic bands (P%) at 99%, number of different genotypes (NG), resolving power (Rp), number of exclusive bands (NEB) and polymorphic information content (PIC).

Primer	TNB	NPB (99%)	P% (99%)	NG	Rp	NEB	PIC
UBC810	12	12	100	11	8.8571	0	0.4596
UBC825	15	14	93	9	9.3333	1	0.3888
UBC815	10	10	100	10	8.0000	0	0.4680
UBC808	13	10	77	6	7.1429	0	0.3279
UBC809	13	12	92	9	8.3810	0	0.3677
UBC850	7	7	100	7	4.2500	0	0.4107
UBC823	8	5	63	10	3.3333	0	0.2642
TOTAL	78	70				1	
Average	11		89	9	7.0425	0.1	0.3838

**Table 2:** Species, geographical coordinates and herbarium where the samples were deposited.

Species	Name	Province	Latitud (S)	Longitud (W)	Herbarium ^1^
*P. tamarugo*	Pt1	El Tamarugal	20°19′46.1″	69°42′27.2″	EIF 13338
*P. tamarugo*	Pt2	El Tamarugal	20°20′37.1″	69°39′53.2″	EIF 13337
*P. tamarugo*	Pt3	El Tamarugal	20°20′57.5″	69°39′51.1″	EIF 13336
*P. tamarugo*	Pt4	El Tamarugal	20°21′03.6″	69°39′48.1″	EIF 13335
*P. tamarugo*	Pt5	El Tamarugal	20°21′03.6″	69°39′47.9″	EIF 13334
*P. tamarugo*	Pt6	El Tamarugal	20°21′22.5″	69°39′12.9″	EIF 13333
*P. burkartii*	Pb1	El Tamarugal	20°23′11.5″	69°35′57.3″	EIF 13344
*P. burkartii*	Pb2	El Tamarugal	20°27′59.2″	69°33′23.2″	EIF 13347
*P. burkartii*	Pb3	El Loa	22°59′3.29″	68°9′19.23″	EIF 13824
*P. burkartii*	Pb4	El Tamarugal	20°28′00.1″	69°33′23.2″	EIF 13348
*P. burkartii*	Pb5	El Tamarugal	20°24′45.0″	69°41′29.7″	EIF 13355
*P. strombulifera*	Ps1	El Tamarugal	20°27′59.8″	69°33′23.5″	EIF 13332
*P. strombulifera*	Ps2	El Tamarugal	20°28′00.1″	69°33′23.3″	EIF 13351
*P. strombulifera*	Ps3	El Tamarugal	20°27′59.9″	69°33′23.5″	EIF 13350
*P. strombulifera*	Ps4	El Tamarugal	20°28′00.2″“	69°33′23.3″	EIF 13352
*P. strombulifera*	Ps5	El Tamarugal	20°30′09.7″“	69°22′54.1″	EIF 13822
*P. reptans*	Pr1	Copiapó	27°20′10.1″	70°35′42.8″	EIF 13324
*P. reptans*	Pr2	Copiapó	27°20′10.9″	70°35′42.7″	EIF 13331
*P. reptans*	Pr3	Copiapó	27°20′10.7″	70°35′42.6″	EIF 13325
*P. reptans*	Pr4	Copiapó	27°20′06.3″	70°35′45.1″	-------------
*P. reptans*	Pr5	Copiapó	27°20′08.6″	70°35′42.8″	EIF 13326
*P. flexuosa*	Pf	Copiapó	27°21′21.8″	70°39′54.7″	EIF 13330
*P. chilensis*	Pc	Chacabuco	33°05′24.9″	70°39′07.4″	EIF 13328
*P. alba*	Pa	Copiapó	27°21′39.3″	70°20′33.8″	EIF 13329

^1^ Index Herbariorum code = EIF.
